# Inducible Lentivirus-Mediated siRNA against TLR4 Reduces Nociception in a Rat Model of Bone Cancer Pain

**DOI:** 10.1155/2015/523896

**Published:** 2015-10-18

**Authors:** Ruirui Pan, Huiting Di, Jinming Zhang, Zhangxiang Huang, Yuming Sun, Weifeng Yu, Feixiang Wu

**Affiliations:** ^1^Department of Anesthesiology, Eastern Hepatobiliary Hospital, Second Military Medical University, Shanghai 200438, China; ^2^Department of Anesthesiology, Kunming General Hospital of Chengdu Military Command, Yunnan 650032, China

## Abstract

Although bone cancer pain is still not fully understood by scientists and clinicians alike, studies suggest that toll like receptor 4 (TLR4) plays an important role in the initiation and/or maintenance of pathological pain state in bone cancer pain. A promising treatment for bone cancer pain is the downregulation of TLR4 by RNA interference; however, naked siRNA (small interference RNA) is not effective in long-term treatments. In order to concoct a viable prolonged treatment for bone cancer pain, an inducible lentivirus LvOn-siTLR4 (tetracycline inducible lentivirus carrying siRNA targeting TLR4) was prepared and the antinociception effects were observed in bone cancer pain rats induced by Walker 256 cells injection in left leg. Results showed that LvOn-siTLR4 intrathecal injection with doxycycline (Dox) oral administration effectively reduced the nociception induced by Walker 256 cells while inhibiting the mRNA and protein expression of TLR4. Proinflammatory cytokines as TNF-*α* and IL-1*β* in spinal cord were also decreased. These findings suggest that TLR4 could be a target for bone cancer pain treatment and tetracycline inducible lentivirus LvOn-siTLR4 represents a new potential option for long-term treatment of bone cancer pain.

## 1. Introduction

Cancer-induced bone pain, characterized by spontaneous pain, hyperalgesia, and allodynia, is estimated to affect about 36%–50% of cancer patients [1]. Severe and long-lasting pain brings agonies to people's daily life, especially for those terminal cancer patients [2]. However, the treatment of bone cancer pain remains a clinical challenge. New methods are urgently needed for this worldwide problem.

Bone cancer pain is considered to be mechanistically unique compared with inflammatory or neuropathic pain states [3]. The activation and upregulation of glial cells in the spinal cord play an important role in initiation and/or maintenance of pathological pain state in bone cancer pain [4, 5]. One of the neuron-to-glial activation signals has proposed that proinflammatory cytokines, such as IL-1, IL-6, and TNF-*α*, were released via the microglial TLR4 receptor in a rat model of bone cancer pain [6]. Administration of a potent TLR4 antagonist (FP-1) reduced both thermal hyperalgesia and mechanical allodynia in the chronic constrictive injury (CCI) models of mice [7]. Thus, blocking the TLR4 signaling pathway might be a useful way of treating bone cancer pain.

RNA interference (RNAi) technique, a promising and potent gene-silencing method, has demonstrated a clinical potential of treating chronic pain by synthetic small interfering RNAs (siRNAs) or short hairpin RNAs (shRNAs) [8]. The key point of this technique is to obtain small interfering RNAs with high “knockdown” efficiency. In the previous work, we screened siRNA sequence with reporter vector and obtained a siRNA against TLR4 with high efficiency [9]. Intrathecal injection of this TLR4 siRNA reduced nociception in a neuropathic pain model of rats [9]. However, the antinociception only lasted for 4 days with naked siRNA. For prolonged antinociception effect, a lentiviral system was addressed in the present study. Meanwhile, for controlling of the targeted gene expression, a tetracycline-regulated gene expression (Tet-on) system was addressed to regulate the expression of small RNA of TLR4. In this system, the targeted gene expression is turned on under the control of doxycycline or tetracycline (Tet). Thus, the antinociception of siRNA expressed by inducible lentivirus was detected in a bone cancer pain model of rats and the inducible effect of doxycycline was observed.

## 2. Materials and Methods

### 2.1. Production and Identification of Inducible Lentivirus LvOn-siTLR4

The siRNA (GUCUCAGAUAUCUAGAUCU) against TLR4 gene (GenBank accession NM_019178) was screened and tested as described in our previous study [9]. Based on the “Tuschl” principle and the sequences of the inducible lentiviral vector, target sequences were designed and chemically synthesized in United Gene Company (Shanghai, China). The target sequences were then cloned into plasmid pLenR-TRIP and named pLenR-TRIP-TLR4. To produce recombinant inducible lentivirus LvOn-siTLR4 (lentivirus expressing siRNA of TLR4), pRsv-REV (20 *μ*g), pMDlg-pRRE (15 *μ*g), and pMD2G (10 *μ*g) were cotransfected into HEK 293T cells with Lipofectamine 2000 [10, 11]. About 48 h after transfection, the lentivirus was harvested. The final titer of recombinant lentivirus was adjusted to 1 × 10^9^ TU/mL.

### 2.2. Induction of Bone Cancer

Bone cancer was induced by Walker 256 cells (breast tumor cells) as we previously described [12]. The left leg was shaved and the skin was disinfected with 70% (v/v) ethanol, after rats were anesthetized by intraperitoneal administration of sodium pentobarbital (40 mg/kg). A rostrocaudal incision of 1 cm was made in the skin over the lower one-third of the tibia for clear exposure with minimal damage to nerves and muscles. The medullary canal was approached by inserting a 23-gauge needle proximally through a hole drilled in the tibia. The needle was then replaced with a 20 *μ*L microinjection syringe. A 10 *μ*L volume of Walker 256 cells (2 × 10^5^ cells) or boiled cells (sham group) was injected into the bone cavity. The syringe was removed after a 2 min delay allowing cells to fill the cavity. The drill hole was sealed with bone wax and the wound was closed using 1-0 silk threads. The rats were allowed unrestricted movement in the cages after recovery and the general condition of rats was monitored during the experiment.

### 2.3. Lumbar Subarachnoid Catheterization

Rats were anesthetized with sodium pentobarbital (40 mg/kg, i.p.). A PE-10 catheter (Becton Dickinson, Sparks, MD, USA) was inserted into the lumbar subarachnoid space between lumbar vertebrae 5 (L5) and L6 [13]. The catheter was chronically implanted and the external part of the indwelling catheter was protected according to Milligan's method [14]. A lidocaine test was given to determine the functionality and position of the catheter tip in the subarachnoid space.

### 2.4. Intrathecal Delivery of Lentivirus

Rats were randomly divided into 6 groups (*n* = 60 per group): a sham group (sham surgery + normal saline), a normal saline (NS) group (cancer + NS), an Lv-MM group (cancer + Lv-MM), an LvOn-siTLR4 group (cancer + LvOn-siTLR4 + NS), a Dox group (cancer + Dox), and an LvOn-siTLR4 with Dox group (cancer + LvOn-siTLR4 + Dox). Lentivirus Lv-MM expressing scrambled siRNA (TTCTCCGAACGTGTCACGT) was used as a control. Four days after cancer cells injection, rats in the LvOn-siTLR4 group and LvOn-siTLR4 with Dox group were given the virus LvOn-siTLR4 (1 × 10^7^ TU/10 *μ*L), respectively. In the Lv-MM group, the same titer of the lentivirus Lv-MM was given intrathecally as a control. The normal saline of equal volume was administered intrathecally in rats of remaining 3 groups. In the Dox group and LvOn-siTLR4 with Dox group, doxycycline was given orally.

### 2.5. Mechanical Allodynia Test

To assess mechanical hyperalgesia, animals were acclimated daily for 10 min/day to the test environment during 3 days, which was a Plexiglass box on a metal grid surface. On test days, rats were allowed to acclimate for 5–10 min. The nociceptive stimulus, a single rigid filament attached to a hand-held transducer (Electronic von Frey Anesthesiometer; IITC, Woodland Hills, CA), was applied perpendicularly to the medial surface of the hind paw with increasing force. The endpoint was taken as nocifensive paw withdrawal accompanied by head turning, biting, and/or licking. As soon as this reaction occurred, the required pressure was indicated in grams, and this value was considered to be the individual paw withdrawal threshold (PWT) value. Each rat was tested in triplicate per time point and the average for the three measurements was then calculated.

### 2.6. Spinal Cord RNA Extraction and Real-Time PCR

The real-time PCR was performed on the 1st, 3rd, 7th, 14th, and 21st days after intrathecal injection of the virus. Total RNA (6 samples of each group) was extracted from L4-L5 spinal cord. Extracted RNA was treated with DNase I at 37°C for 30 min before reverse transcription was performed using a kit (TaKaRa, Japan). The PCR primers were as follows: 5′-CGGGAG CTC TGA ATG CTC TCT TGC ATC TGG CTG GC-3′ (forward) and 5′-CGG GTC GAC GCC ATA CAA TTC GACCTG CTG-3′ (reverse). The Real-Time PCR Detection System (Roche, Switzerland) continually monitors the increase in fluorescence, which is directly proportional to the PCR product [15].

### 2.7. Western Blot Assay

The proteins of tissues from lumbar spinal cord (L4-L5) were prepared on the 7th day after injection as previously described [16]. Proteins were separated by 8% polyacrylamide SDS-PAGE and transferred onto a nitrocellulose membrane. The nitrocellulose membrane was blotted with a primary antibody against TLR4 (1 : 100, Santa Cruz, USA) and then with secondary antibody conjugated with horseradish peroxidase. Protein signals were detected with an ECL system (Amersham Pharmacia, Uppsala, Sweden). GAPDH (Sigma Chemical Co., MO, USA, 1 : 500) was used as a loading control. The images were captured and analyzed by ImageJ software.

### 2.8. Enzyme Linked Immunosorbent Assay (ELISA)

To detect TNF-*α* and IL-1*β* proteins, samples from the spinal cord (L4-L5) were analyzed by ELISAs specific for these cytokines. The samples were prepared on the 1st, 3rd, 7th, 14th, and 21st days after intrathecal injection of the virus as previously described [17]. The ELISAs for TNF-*α* and IL-1*β* in the spinal tissue were performed according to the manufacturer's instructions (Peprotech, UK). Total protein concentrations of TNF-*α* and IL-1*β* were determined by the Bradford assay and used to adjust results for sample size [18].

### 2.9. Statistical Analysis

All data were expressed as mean ± standard error (SEM). Statistical analysis was carried out using two-way ANOVA followed by Tukey's multiple comparisons using GraphPad Prism software (Version 5. GraphPad Software Inc., CA, USA). The data from western blotting was compared using one-way ANOVA. *P* < 0.05 was considered statistically significant.

## 3. Results

### 3.1. LvOn-siTLR4 with Dox Attenuated Bone Cancer Pain

**Figure 1:**
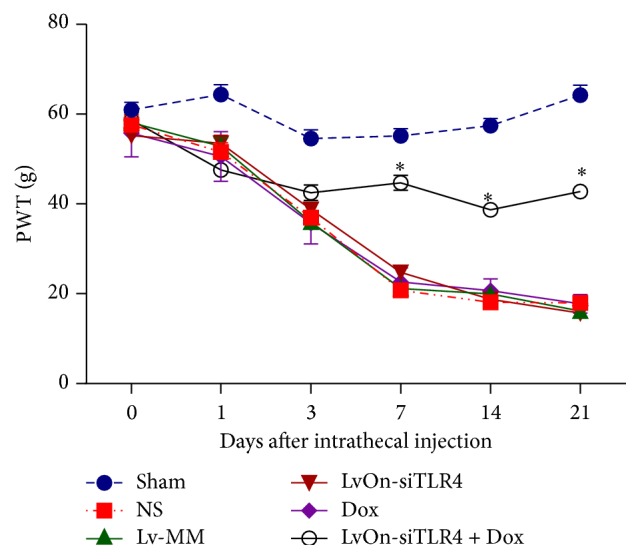
Impact of LvOn-siTLR4 with Dox on PWT in bone cancer pain rats. The bone cancer pain was set up by the injection of Walker 256 cells into the legs of rats. After cancer cells injection, rats received intrathecal administration of the virus on the 4th day. On the 7th, 14th, and 21st days after intrathecal administration of the virus, bone cancer pain rats receiving intrathecal LvOn-siTLR4 with oral administration of doxycycline showed significantly attenuated mechanical allodynia compared to the rats treated with Lv-MM, normal saline, doxycycline, and LvOn-siTLR4 on PWT (^*∗*^ *P* < 0.01 versus Lv-siTLR4 group, Lv-MM group, NS group, and Dox group, two-way ANOVO analysis followed by Tukey's multiple comparisons, *N* = 10).

To examine the impact of inducible lentivirus LvOn-siTLR4 on pain response* in vivo*, modulation of pain perception in the bone cancer pain model was investigated. PWT was used to measure the mechanical allodynia. After surgery, mechanical allodynia was induced, in correspondence with the reduced PWT. Compared with that in the sham group, mechanical allodynia significantly increased in the rats receiving Walker 256 cells injection (*P* < 0.01, *N* = 10, Figure 1). In contrast to the Lv-MM group and NS group, mechanical allodynia was decreased in the LvOn-siTLR4 with DOX group on the 3rd, 7th, 14th, and 21st days after viral injection (*P* < 0.01), which suggested that the small RNA expressed by the lentivirus was effective. Meanwhile, in the LvOn-siTLR4 group, rats without Dox had no effect in mechanical allodynia, which illustrated that the expression of small RNA was induced by Dox. Moreover, PWT did not change in the Dox group compared to that in the NS group, suggesting that Dox did not contribute to the mechanical allodynia. The process lasted for about 21 days, which indicated that the antinociception effect of LvOn-siTLR4 was long-lasting.

### 3.2. LvOn-siTLR4 with DOX Decreased TLR4 Expression

**Figure 2:**
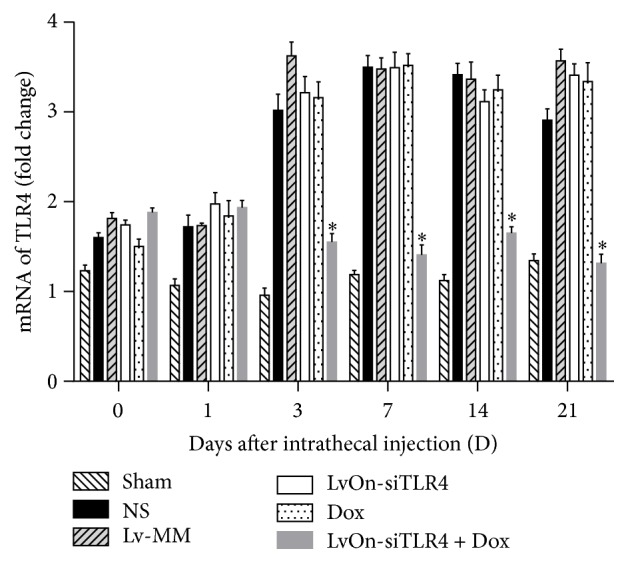
mRNA expression of TLR4 detected by real-time PCR. As shown in Figure 2, bone cancer in legs increased the TLR4 mRNA expression in the spinal cord in the five groups receiving Walker 256 cells injection. On the 3rd, 7th, 14th, and 21st days after delivery of Lv-siTLR4 with oral administration of doxycycline, the TLR4 mRNA expression decreased significantly in the Lv-siTLR4 with Dox group compared the other four groups receiving Walker 256 cells injection (^*∗*^ *P* < 0.01 versus Lv-siTLR4 group, Lv-MM group, NS group, and Dox group, two-way ANOVO analysis followed by Tukey's multiple comparisons, *N* = 6). No differences were observed on the 1st day after injection.

**Figure 3:**
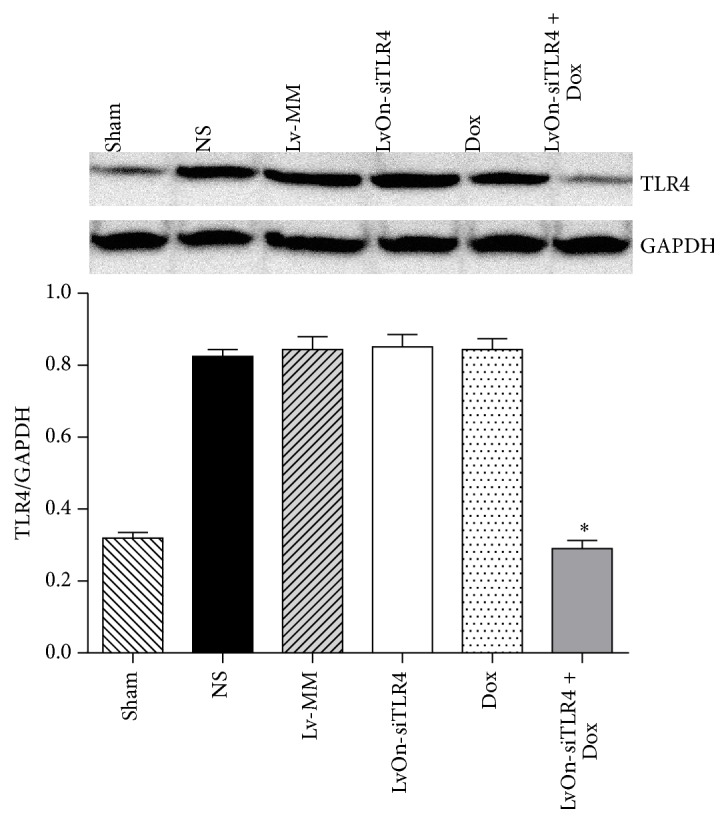
Western blot assay of TLR4 expression. On the 7th day after viral injection, the protein of lumbar spinal cord was prepared and the expression of TLR4 was detected. The protein expression of TLR4 in the Lv-siTLR4 with Dox group was also markedly downregulated compared to the other four groups receiving Walker 256 cells injection (^*∗*^ *P* < 0.01 versus Lv-siTLR4 group, Lv-MM group, NS group, and Dox group, one-way ANOVA analysis, *N* = 6).

LvOn-siTLR4 with DOX was intrathecally delivered into the rats with bone cancer pain and protein expressions of TLR4 and its mRNA were detected. As shown in Figure 2, TLR4 mRNA expression was increased significantly in the rats which received Walker 256 cells injection compared with that in the sham group (*P* < 0.01, *N* = 6). Similar results were shown in the protein expression, which suggested that TLR4 increased in the bone cancer pain models (Figure 3). TLR4 and its mRNA expressions were decreased in the LvOn-siTLR4 with DOX group compared with that in other four groups of bone cancer pain, suggesting that lentivirus expressed small RNA of TLR4 interfered TLR4 expression.

### 3.3. LvOn-siTLR4 with DOX Decreased TNF-*α* and IL-1*β*

**Figure 4:**
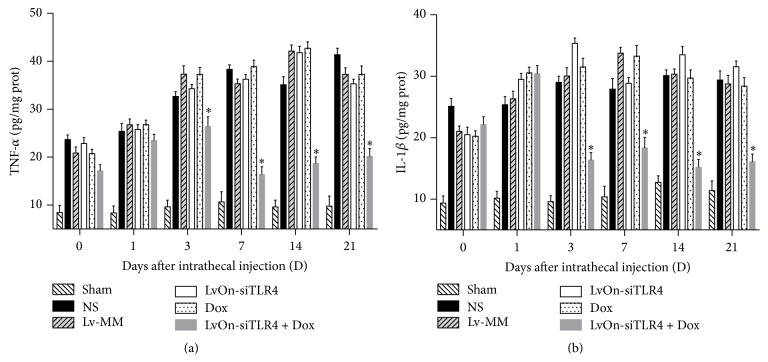
Effects of LvOn-siTLR4 with Dox on proinflammatory cytokine in spinal cord. The dorsal spinal cord tissue samples were prepared on the 1st, 3rd, 7th, 14th, and 21st days after intrathecal injection of the virus for the detection of TNF-*α* and IL-1*β* expression. A significant increase of TNF-*α* (a) and IL-1*β* (b) in the spinal cord was shown in the five groups receiving Walker 256 cells injection. On the 3rd, 7th, 14th, and 21st days after delivery of Lv-siTLR4 with oral administration of doxycycline, the TNF-*α* (a) and IL-1*β* (b) expression decreased markedly in the Lv-siTLR4 with Dox group compared to the other four groups (^*∗*^ *P* < 0.01 versus Lv-siTLR4 group, Lv-MM group, NS group, and Dox group, two-way ANOVA analysis followed by Tukey's multiple comparisons, *N* = 6). No differences were observed on the 1st day after injection.

To investigate whether the antiallodynia effects of the inducible lentivirus were associated with decreased production or release of proinflammatory cytokines, protein levels of TNF-*α* and IL-1*β* were assessed in the spinal cord. As shown in Figures 4(a) and 4(b), rats with bone cancer pain markedly induced upregulation of TNF-*α* and IL-1*β* (*P* < 0.01 versus sham group). In the LvOn-siTLR4 with Dox group, small RNA expressed by the LvOn-siTLR4 significantly inhibited the release of IL-1*β* and TNF-*α* in the spinal cord (*P* < 0.01 versus other four groups). These results indicated that TLR4 small RNA expressed by the virus LvOn-siTLR4 with DOX could disrupt the release of TNF-*α* and IL-1*β*.

## 4. Discussion

In this study, the effects of nociception on bone cancer pain models were investigated by inducible lentivirus expressing siRNA against TLR4. Based on our results, not only could LvOn-siTLR4 attenuate mechanical allodynia by intrathecal injection, but also its induction significantly downregulated mRNA and protein expression of TLR4 in the spinal cord. IL-1*β* and TNF-*α* release were significantly inhibited by small RNA expressed by LvOn-siTLR4 as well. In addition, the small RNA expression by LvOn-siTLR4 could be induced by oral administration of doxycycline. Our findings suggest that the lentivirus-mediated siRNA against TLR4 may be used for gene therapy of bone cancer pain in an experimental setting. If this is successful, the downregulation of TLR4 expression by inducible lentivirus LvOn-siTLR4 may be used to treat bone cancer pain in clinic.

It has been reported that the glial activation in the spinal cord is involved in bone cancer pain [19]. The activated glia and subsequent release of proinflammatory mediators have been implicated in initiating and maintaining pain response. TLR4 has shown mechanistic links between glial activation, innate immunity, and the initiation of behavioral hypersensitivity [20]. Through the activation of TLR4-MyD88-dependent or -independent pathways [21, 22], TLR4 activates intracellular signal molecules including TNF receptor-associated factor 6 and IKK*α*, *β* (I*κ*B kinase) to form a complex and phosphorylated I*κ*B [23]. The phosphorylation leads to the degradation of I*κ*B and subsequent translocation of NF-*κ*B, which controls the releasing of proinflammatory cytokines including TNF-*α* and IL-1*β* [24]. The downregulation of TLR4 by small RNA leading to decrease in the TNF-*α* and IL-1*β* was confirmed in the present study, which was consistent with the former research where blocking the TLR4 decreased the expression of TNF-*α* and IL-1*β* [15]. Activation of the TLR4 pathway in the spinal cord is proven to contribute to the neuropathic pain [7, 25, 26] and the suppression of TLR4 protein expression by siRNA could alleviate hyperalgesia and allodynia in CCI model of rats [9]. The present study showed the reduction of TLR4 and attenuation of allodynia after intrathecal injection of the lentivirus expressing small RNA. It is consistent with the former report that tactile allodynia in rats with bone cancer could be attenuated by the blocking TLR4 [6]. All these suggested that TLR4 signal pathway may involve bone cancer pain and that attenuation of TLR4 may probably be a new treatment of bone cancer pain.

In the application of RNAi technique, the efficiency, specificity, and stability of siRNA in target cells should be considered [27, 28]. Our previous work has showed the specificity and efficiency of small RNA in targeting of TLR4 [15]. However, naked siRNA mediated downregulation of gene expression is not stable and only lasts for 3 to 5 days [9]. Hence, the lentivirus, capable of expressing the target gene for several months and suitable for bone cancer pain treatment [29, 30], was introduced as a tool for siRNA. As hypothesized, results showed that the antinociception effect lasted for about 21 days, which provided a suitable tool for the treatment of bone cancer pain as a chronic state. Our previous work has shown that the lentivirus was successfully transfected into dorsal horn of rats after intrathecal injection [31], which was consistent with the location of TLR4 [32]. The results showed that LvOn-siTLR4 intrathecal injection along with oral administration of doxycycline attenuated allodynia of rats, which suggested that the lentivirus LvOn-siTLR4 could suppress nociception in the bone cancer models through decreasing the expression of TLR4.

For further clinical usage, the levels and timing of TLR4 expression need to be regulated to prevent overreduction of target gene. In the current research, tetracycline-regulated gene expression (Tet-on) system was introduced into the lentivirus. The tetracycline (tet) repressor was fused to a herpes simplex virus (HSV) VP 16 transactivation domain in this system to form a reverse tet-controlled transcriptional activator (rtTA) [33, 34]. In this case, in the presence of Dox or Tet, rtTA is able to bind operator sequences to activate transcription, turning on the target gene expression [35, 36]. Our results showed that TLR4 and its mRNA expressions were decreased after injection of the virus LvOn-siTLR4 with oral administration of Dox, while rats without Dox had no effect on TLR4 and its mRNA. The results illustrated that the expression of small RNA by LvOn-siTLR4 was under the control of Dox. In this case, the timing and levels of TLR4 could be controlled precisely by oral administration of doxycycline to maintain protein concentrations within a therapeutic window for further clinical practice.

However, TLR4 expression regulated by oral administration of doxycycline in a dose-dependent manner is needed. Further researches are warranted.

## 5. Conclusion

In the present study, we showed that downregulation of TLR4 by intrathecal injection of inducible lentivirus LvOn-siTLR4 could prevent bone cancer-induced tactile allodynia and that TLR4 expression could be controlled by doxycycline in regulation of the lentivirus. Our study provided a new approach of bone cancer pain treatment by downregulation of TLR4 expression using an inducible lentivirus LvOn-siTLR4.
